# Is Serum Ferritin an Early Marker for COVID-19-Associated Mucormycosis?

**DOI:** 10.7759/cureus.36734

**Published:** 2023-03-27

**Authors:** Indu DP, Kala Yadhav ML, Chetana GS

**Affiliations:** 1 Microbiology, Shri Atal Bihari Vajpayee Medical College and Research Institution, Bengaluru, IND

**Keywords:** fungal culture, mucorales, serum ferritin, mucormycosis, covid-19

## Abstract

Background

Coronavirus disease 2019 (COVID-19) is a hyperinflammatory disease caused by severe acute respiratory syndrome coronavirus 2, which makes critically ill patients susceptible to invasive fungal infections. Invasive fungal infections such as mucormycosis are associated with high morbidity and mortality. This study aimed to determine the serum ferritin levels in COVID-19-associated mucormycosis (CAM) patients and isolate and identify the fungi causing secondary fungal infections in patients with suspected CAM.

Methodology

This cross-sectional study was conducted from June to September 2021 among CAM patients admitted to Bowring and Lady Curzon Hospital. After obtaining approval from the institutional ethics committee and valid consent, data regarding demographic details, past medical history, and serum ferritin levels, along with other blood investigations, were carefully collected from patients presenting with clinical features of mucormycosis and a history of COVID-19. Samples were examined under a bright field microscope using wet mounts of samples in KOH, cultured on Sabouraud’s dextrose agar, and examined under a microscope after staining with lactophenol cotton blue for the isolation and correct identification of fungi. Statistical analysis was done using Microsoft Excel (Microsoft Corp., Seattle, WA, USA) and SPSS version 26.0 (IBM Corp., Armonk, NY, USA). A p-value less than <0.05 was considered statistically significant.

Results

A total of 95 patients with CAM were included in this study, comprising 70 males and 25 females. The mean age of presentation was 49.83 ± 12.41 years, with 73% males and 26% females. Type 2 diabetes mellitus was noted in 69% of patients, hypertension in 29%, and steroid use in 42%. The mean serum ferritin level was 537.38 ± 468.88 ng/mL. We found a significant association between increased serum ferritin and a history of diabetes. Serum ferritin levels had a statistically significant correlation with samples of patients who were positive for Mucorales under KOH microscopy. The fungal culture showed the growth of *Aspergillus*, *Mucor*, *Rhizopus*, and *Candida*. The mean value of serum ferritin in patients who showed mucor growth was 842.09 ng/mL.

Conclusions

We found a significant increase in serum ferritin levels in CAM patients. Ferritin can be used as an early marker for screening mucormycosis in COVID-19 patients. Monitoring patients with elevated serum ferritin levels in severe COVID-19, glycemic control, judicious use of corticosteroids, early diagnosis, and appropriate treatment can aid in better management of the disease.

## Introduction

​​​​The coronavirus disease 2019 (COVID-19) caused by severe acute respiratory syndrome coronavirus 2 (SARS-CoV-2) was declared a pandemic by the World Health Organization on March 12, 2020 [1]. COVID-19 exhibits a broad variety of clinical symptoms, ranging from asymptomatic infection to severe pneumonia, followed by multisystemic failure that can result in death [2]. Impaired cell-mediated immune response, elevated systemic inflammatory markers and cytokines, and reduced CD4+ T and CD8+ T cells seen in COVID-19 patients make them susceptible to invasive fungal infections [3]. Mucormycosis is one such opportunistic fungal infection seen secondary to COVID-19 infection.

Mucormycosis is an aggressive, angioinvasive, opportunistic fungal infection caused by fungi of the order of Mucorales belonging to the class of zygomycetes [4]. Mucormycosis is acquired primarily via inhalation of environmental sporangiospores in immunocompromised hosts [4]. Common species causing mucormycosis include *Rhizopus *spp., *Mucor* spp., *Lichtheimia* spp., *Apophysomyces* spp., *Cunninghamella* spp., and *Saksenaea *spp. *Rhizopus arrhizus *is the predominant agent causing COVID-19-associated mucormycosis (CAM) in India [4]. India has a prevalence of 14 cases per 100,000 population with a mortality of 38%, which is nearly 70 times the global prevalence of 0.2/100,000 [5].

Mucormycosis is classified according to the anatomical site of infection as rhino-cerebral, rhino-orbital, pulmonary, cutaneous, gastrointestinal, disseminated, or other uncommon presentations, such as endocarditis, osteomyelitis, peritonitis, and renal infection [6]. Rhino-orbital mucormycosis is the most common presentation (42%), followed by rhino-orbito-cerebral mucormycosis (24%) and pulmonary mucormycosis (10%) [7]. Predisposing risk factors for mucormycosis include uncontrolled diabetes, use of systemic corticosteroids, neutropenia, hematologic malignancies, stem cell transplant, and immunocompromised individuals [8]. Iron overload is seen in all these conditions, which is a prerequisite for the virulence of invasive fungal species [9,10].

Ferritin is an iron-binding protein that is elevated in conditions of iron overload and inflammation. It plays a critical role in iron homeostasis by iron sequestration [11]. The events of the release of pro-inflammatory cytokines, such as interleukin (IL)-6, IL-1, and tumor necrosis factor-alpha (TNF-α), increased ferritin, iron toxicity, cellular damage, metabolic acidosis, free radical generation, and secondary tissue damage lead to the availability of free iron to zygomycetes [12]. Fungi can obtain iron from the host by using siderophores, rhizoferrin, heme oxygenase homologs, and other genes which help in adaptation to ambient iron availability [10].

The catastrophic outburst of cases of CAM during the second wave of the COVID-19 pandemic warranted studies to determine the possible pathogenic mechanisms and risk factors to aid in better management of the disease. With the established importance of iron in the pathogenic mechanism of zygomycetes in the literature, this study was conducted to determine the serum ferritin levels in CAM patients as well as to isolate and identify the fungi causing secondary fungal infections in patients with suspected CAM.

## Materials and methods

This cross-sectional study was conducted from June 2021 to September 2021 among 95 CAM patients admitted to Bowring and Lady Curzon Hospital in Bangalore, Karnataka, India.

Study population

Inclusion Criteria

All clinically and radiologically confirmed mucormycosis patients with a history of reverse transcription-polymerase chain reaction (RT-PCR)-confirmed COVID-19 diagnosis, belonging to the age range of 18-80 years old, and willing to give consent were included in this study.

Patients presenting with facial pain, nasal congestion, nasal ulcer with black eschar, headache, fever, facial edema, ulcer over the palate, toothache, orbital inflammation, retro-orbital cellulitis, changes in ocular motility, blurring of vision, diplopia, proptosis, and CT or MRI scans of the brain and paranasal sinuses showing mucosal thickening, bone erosion, edema, brain inflammation, with present or previous RT-PCR-confirmed COVID-19 diagnosis in nasopharyngeal swabs were included [4].

Exclusion Criteria

Non-consenting patients with no history of COVID-19 and with other conditions associated with high ferritin levels, such as liver dysfunction, rheumatologic diseases, hematological malignancies, renal failure, solid tumors, hemolytic anemia, and HIV infection, were excluded.

After obtaining institutional ethical clearance (reference number: BLCMCRI/IEC/ARP/099/2021-22) and informed consent from the patients, data regarding demographic details, past medical history, serum ferritin levels, and other blood investigations were carefully collected from the case records as per the proforma. COVID-19 was diagnosed using the CoviPath™ COVID-19 RT-PCR kit (manufactured by Invitrogen Bioservices Pvt. Ltd., Bangalore, India). Serum ferritin was analyzed using the automated immunoassay system, the Access Ferritin assay, which uses chemiluminescent immunoassay for the quantitative determination of ferritin levels in human serum (Beckman Coulter, Brea, CA, USA).

Processing of samples

Samples such as nasal crusts, swabs, nasal wash, and tissue biopsy were included for potassium hydroxide (KOH) microscopy, lactophenol cotton blue (LPCB) mount, and Sabouraud’s dextrose agar (SDA) culture.

Homogenization

Samples such as hard tissues were digested using 20-40% KOH overnight. Soft tissues were not digested and were gently teased onto the glass slide for microscopy. Liquid samples were first concentrated by centrifugation and the sediment was used for microscopy and culture.

Direct Microscopy

Wet mounts of samples in KOH were examined under a bright field microscope for aseptate fungal elements.

Culture

Processed samples were inoculated into two SDA tubes with chloramphenicol and sealed. Each tube was incubated at room temperature and at 37°C. The tubes were examined daily for one week, and from the eighth day, they were examined on alternate days.

Lactophenol Cotton Blue Mount

A small portion of growth midway between the colony center and edge was subjected to LPCB mount.

Identification

The Mucorales were identified by their gross colony and microscopic morphology. Broad aseptate hyphae with obtuse angle branching on microscopy, white cottony woolly colonies on SDA at 25°C, and aseptate hyphae ending at sporangium and with nodal rhizoids on LPCB mount were diagnosed as Mucorales.

Data analysis

After the collection and compilation of data, baseline characteristics were tabulated in a licensed Microsoft Excel sheet (Microsoft Corp., Seattle, WA, USA). Data were analyzed using the statistical package SPSS version 26.0 (IBM Corp., Armonk, NY, USA), and the level of significance was set at p-values of <0.05. Descriptive statistics were performed to assess the proportion of the respective groups. The normality of the data was assessed using the Shapiro-Wilk test. Because the data did not follow the normal distribution, non-parametric tests were used for data analysis. Inferential statistics to determine the difference between the groups were done using the Kruskal-Wallis test followed by Tukey’s honestly significant difference (HSD) post hoc analysis to determine the difference between any two groups. Mann-Whitney U test was used to check the difference between the two groups.

## Results

A total of 95 patients who met the inclusion criteria were included in this study. Of these patients, 70 were male and 25 were female, accounting for 73% and 26%, respectively. The mean age of presentation was 49.83 ± 12.41 years, ranging from 28 to 77 years. The mean age of presentation was 49.91 ± 12.08 years in males and 49.6 ± 13.53 years in females.

Most patients belonged to the 41-50-year age group, with fewer cases seen in the 20-30-year age group. Male patients were more common in most age groups, except for the 31-40-year age group, which had an equal representation of both males and females. In the 71-80-year age group, female patients predominated.

**Figure 1:**
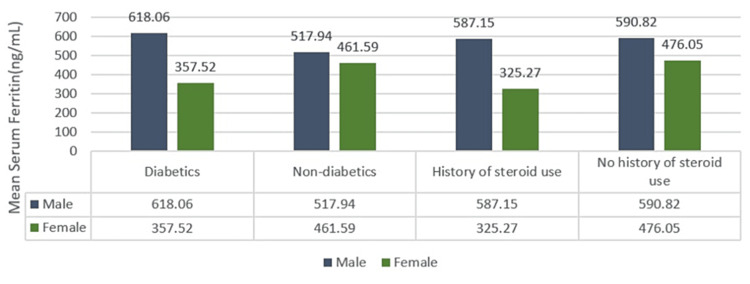
Mean serum ferritin in patients with diabetes mellitus and a history of steroid use.

The most common comorbidities observed were diabetes mellitus, followed by hypertension. Overall, 66 (69%) patients had diabetes mellitus, and 28 (29%) patients had hypertension. Further, 40 (42%) patients had a history of steroid use. The mean serum ferritin value in patients with diabetes was 569.12 ng/mL (Figure 1, Table 1).

**Table 1:** Association of serum ferritin levels with a history of diabetes.

		Mean (in ng/mL)	SD (in ng/mL)	Minimum (in ng/mL)	Maximum (in ng/mL)
Diabetes	Yes	555.03	454.52	23	2,000
	No	499.15	488.99	15.5	1,887.2
P-value		0.005			

**Figure 2:**
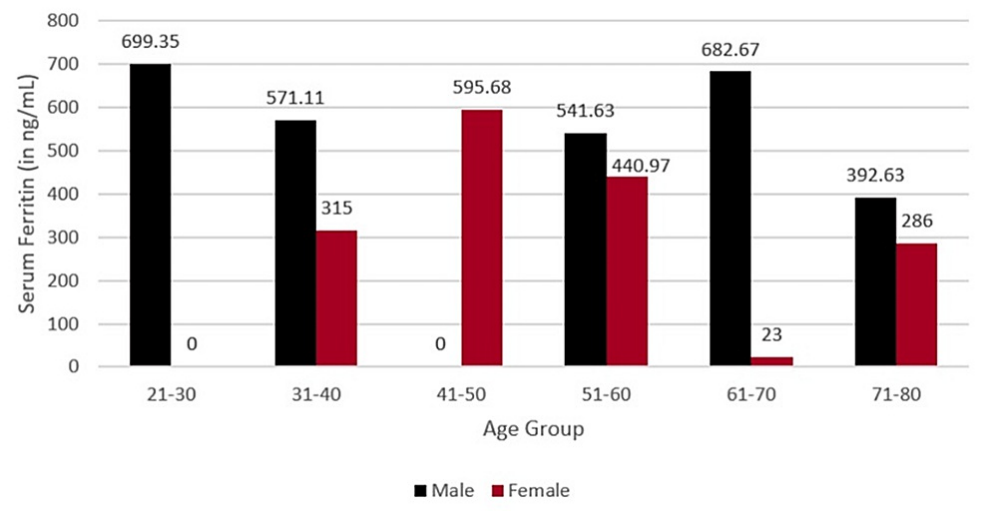
Serum ferritin levels in different age groups.

The mean time interval between COVID-19 diagnosis and suspicion of mucor infection was 45 ± 36.20 days. The levels of ferritin in the patients ranged from 195.3 to 1,993 ng/mL, with a mean of 537.38 ± 468.88 ng/mL. Normal serum ferritin levels were taken as 24-336 ng/mL. The mean value of serum ferritin in males was 589.45 ± 457.93 ng/mL, and in females, it was 391.61 ± 477.66 ng/mL. Elevated levels of serum ferritin were observed in 47 males and 12 females (Figure 2).

Out of the 95 samples collected from patients with suspected CAM, 14 were found to be positive for fungal elements upon KOH examination. Among these samples, 10 (10.52%) showed the presence of broad aseptate hyphae with obtuse angle branching, suggestive of Mucorales. The mean serum ferritin value in patients who were KOH positive for Mucorales was 1,084.43 ng/mL. Our analysis showed a statistically significant correlation between serum ferritin levels and samples that were KOH positive for Mucorales (p < 0.001) (Table 2).

**Table 2:** Association of serum ferritin levels with KOH positives for Mucorales.

KOH	Mean (in ng/mL)	SD (in ng/mL)	Lower bound (in ng/mL)	Upper bound (in ng/mL)	P-value
Positive	1,084.43	529.24	111	2,000	<0.001
Negative	459.87	392.53	15.5	1,993	

A total of 18 samples showed fungal culture growth, with *Aspergillus*, *Mucor*, *Rhizopus*, and *Candida *being the most commonly isolated fungi. Among the nine samples that showed growth for *Aspergillus*, five were identified as *Aspergillus fumigatus*, three were *Aspergillus flavus*, and one was *Aspergillus niger*. The mean value of serum ferritin in samples of patients who showed mucor growth was 842.09 ng/mL. No significant correlation was seen between different fungal genera.

**Figure 3:**
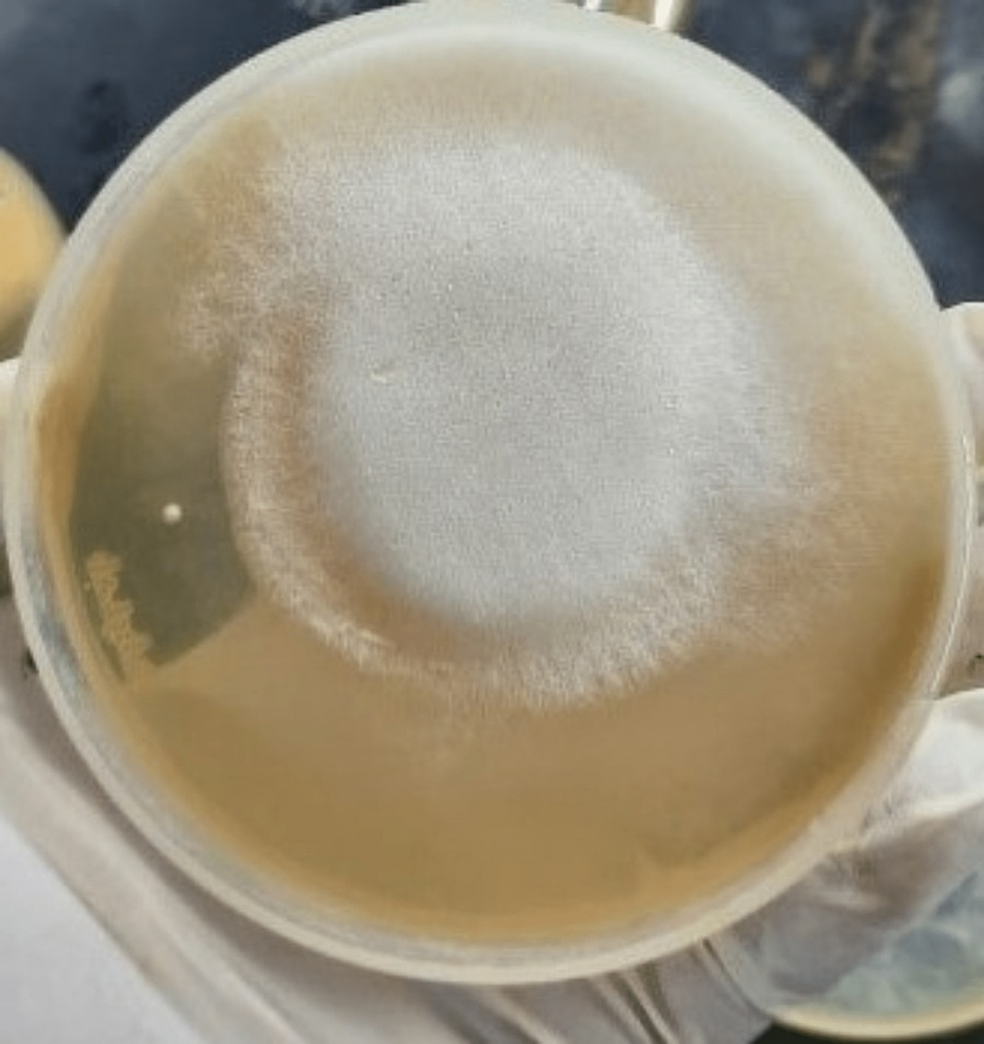
Mucor growth on Sabouraud’s dextrose agar.

**Figure 4:**
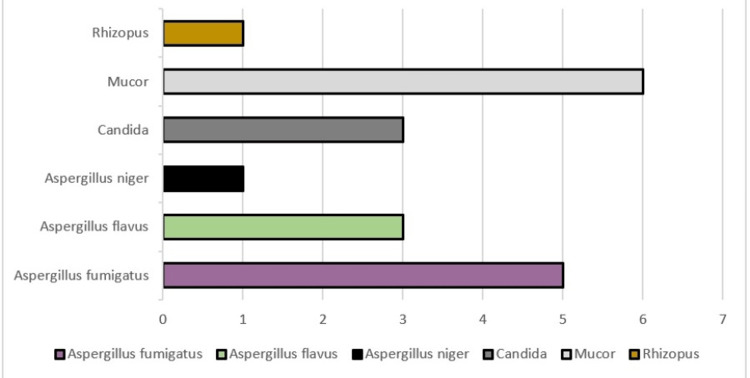
Fungal culture growth.

It is noteworthy that only four samples that were KOH microscopy positive for Mucorales showed fungal culture growth for Mucorales, and two samples that were KOH negative showed mucor growth on culture (Figure 3, Figure 4, and Table 3).

**Table 3:** Mean value of serum ferritin levels in different fungal genera.

Genera isolated	Mean serum ferritin level (ng/mL)	SD	Lower range (ng/mL)	Upper range (ng/mL)
Aspergillus	318.99	274.79	23	905
Mucor	842.09	480.21	261	1,528.1
Candida	522.06	224.68	288	825.2
No growth	502.36	440.96	15.5	1,993

## Discussion

This cross-sectional study aimed to evaluate the serum ferritin levels in a group of 95 patients who were clinically suspected and radiologically confirmed with CAM and aged between 20 and 80 years. Our study revealed a significant elevation in the mean serum ferritin levels among CAM patients. However, we observed a low yield of positive results for KOH microscopy and fungal culture of samples. These findings hold great significance considering the recent outbreak of CAM, as serum ferritin levels can serve as a useful marker for risk assessment of mucormycosis development in COVID-19 patients.

Viremia seen secondary to COVID-19 causes cytokine storm, where there is an excessive release of pro-inflammatory cytokines such as IL-1, IL-6, and TNF-α. This causes iron dysregulation by increasing the production of ferritin and hepcidin. Hepcidin reduces the export of iron from the cell, resulting in iron toxicity, ferroptosis, and the release of iron. Iron is essential for several critical cellular processes, including the transport of oxygen and enzyme function, making it an essential entity for the survival of organisms, much so for zygomycetes [13]. Our research found that the average serum ferritin level was 537.38 ± 468.88 ng/mL. Similar results were reported in a study by Yeshupatham et al. [14] among 84 patients with confirmed fungal infections, where the average serum ferritin level was 528.58 ± 45.03 ng/mL. In a case study of 300 COVID-19 patients with mucormycosis by Goddanti et al. [15], a similar average serum ferritin level of 461.31 ± 26 ng/mL was observed in patients aged 20-90 years.

Our study found that 69.4% of the participants had diabetes mellitus, and 42.1% had a history of steroid use. Similarly, in a study by Bhadania et al. [16], 53.6% of the patients with mucormycosis had diabetes mellitus. In contrast, Yeshupatham et al. [14] reported that 94.1% of their patients had diabetes mellitus, and 70.83% had a history of steroid use. Hyperglycemia can lead to the expression of the glucose-regulated protein (GRP 78) and Mucorales adhesin spore coat protein homologs (CotH) on endothelial cells. This, in turn, increases the adhesion and penetration of Mucorales to the endothelium [17]. Uncontrolled diabetes or steroid use can cause hyperglycemia, which results in the glycosylation of iron-binding proteins such as transferrin and ferritin. This reduces their iron-binding capacity, leading to the release of free iron, which can then be utilized by Mucorales [18].

The gold standard for diagnosing mucormycosis is fungal culture from clinical samples. However, our study found that only 10.52% of the samples were positive for Mucorales under KOH mount, which could be attributed to damage to Mucorales during the sample processing. Lakshmanan et al. [19] also reported a lower KOH positivity of 21.6%. We observed a mean serum ferritin value of 1,084.43 ng/mL in patients who were positive for Mucorales under KOH mount. We found a statistically significant correlation between elevated serum ferritin levels and KOH positivity. Fungal culture revealed the growth of organisms belonging to different genera such as *Aspergillus*, *Mucor*, *Rhizopus*, and *Candida*. This highlights the importance of fungal culture in diagnosing mucormycosis despite its low sensitivity. Fungal culture allows for the identification of the specific fungal pathogen and helps to select appropriate antifungal therapy, which is crucial in preventing the development of antifungal resistance.

In our study, among the other fungal genera, mucor exhibited the highest mean serum ferritin level of 842.09 ng/mL. This finding supports the role of iron in the pathogenesis of mucormycosis, which has been established previously in the literature. Ibrahim et al. [20] discussed iron acquisition and metabolism in fungi, which occurs through various mechanisms such as high-affinity iron permeases or siderophores that aid in reducing ferric to ferrous ions, rhizoferrin that helps in capturing iron, and heme oxygenase homologs that contribute to the angioinvasive nature of Mucorales.

The major limitation of our study is its cross-sectional design. A case-control study design would have been more suitable for investigating the association between elevated serum ferritin levels and CAM. However, our study had a good sample size of 95 CAM patients and is one of the very few studies to correlate serum ferritin levels with KOH positivity for Mucorales.

Future studies that address the limitations of our study can help validate the association between hyperferritinemia and CAM. Despite the limitations, our study provides useful insights into the pathogenesis and diagnosis of this potentially fatal fungal infection in COVID-19 patients.

## Conclusions

A considerable amount of evidence exists in the literature regarding the role of iron in the pathogenesis of mucormycosis. Elevated levels of serum ferritin have been found to be a major factor in the pathogenesis of the disease. Our study confirmed a significant increase in serum ferritin levels in CAM patients, indicating its relevance as an early marker for assessing the risk of mucormycosis.

Furthermore, we found that COVID-19 patients with uncontrolled diabetes and those who had received steroid treatment had a higher risk of developing mucormycosis. Hyperglycemia due to uncontrolled diabetes or steroid use can lead to glycosylation of iron-binding proteins, such as transferrin and ferritin, thereby reducing their iron-binding capacity and releasing free iron to Mucorales. This iron can further contribute to the development of the disease.

Monitoring CAM patients with abnormally elevated serum ferritin levels and maintaining a high index of clinical suspicion, along with the control of hyperglycemia and judicious use of steroids, can aid in better management of the disease.
